# Ultrasound findings of the thyroid gland in children and adolescents

**DOI:** 10.1007/s40477-022-00660-9

**Published:** 2022-02-09

**Authors:** Elena Moschos, Hans-Joachim Mentzel

**Affiliations:** 1 Institute of Diagnostic and Interventional Radiology, University Hospital Jena, Am Klinikum 1, 07747 Jena, Germany; 2 Section of Paediatric Radiology, Institute of Diagnostic and Interventional Radiology, University Hospital Jena, Am Klinikum 1, 07747 Jena, Germany

**Keywords:** Thyroid disease, Children, Ultrasonography, Elastography, CEUS

## Abstract

Ultrasonography (US) is an important diagnostic tool in evaluating thyroid diseases in pediatric patients. This pictorial essay reviews the application of various ultrasound techniques such as B-Mode ultrasound and color Doppler, elastography and contrast enhanced ultrasound (CEUS) in children and adolescents in various thyroid pathologies including congenital thyroid abnormalities, diffuse thyroid diseases (DTD), focal thyroid lesions and thyroid malignancy.

## Key points

**Table:** 

Early diagnostic of thyroid disease in children and adolescents is important for their growth and development.
Ultrasonography plays an important role in evaluating thyroid disease.
Combination of various ultrasound techniques can help better evaluate thyroid pathology.

## Introduction

Thyroid disease is a growing problem in children and adolescents [1]. Its early diagnosis is important for growth and development in this age group. High-resolution ultrasound combined with clinical examination and function tests are currently the preferred methods to evaluate the thyroid [2]. In the recent years there have been more and more publications on new US diagnostic imaging tools, such as elastography and thyroid CEUS in adults [3–6]. Up to now there is no overview about the possibilities of these new modalities in children and adolescents.

We describe some sonographic findings in thyroid disease in children and adolescents using various US methods.

## Technique

For ultrasound examination the patient is in a supine position with extended neck with a bolster or folded towel under his shoulders. The transducer is placed in a transverse plane over the anterior neck area. Sometimes a gel wedge is used to achieve an ideal coupling of the probe and to avoid artifacts. The transducer then should be slided the entire embryological path of the thyroid from the sternal notch up to the mandible. A scan should include pictures of the thyroid and isthmus in both transverse and longitudinal planes. It is important to evaluate cervical lymph nodes, common carotid artery, and the internal jugular vein as well.

High-frequency linear-array (7- to 18-MHz) and in case of a large struma or in infants with short neck small phased-array or sector transducers (2- to 10-MHz) are used to obtain detailed anatomical information [7]*.* B-Mode ultrasound and color Doppler are considered to be the standard sonographic imaging methods in pediatric population [8]. Grayscale imaging will be used to describe echotexture of diffuse parenchymal abnormalities and focal lesions in comparison to normal thyroid tissue and to submandibular gland. Doppler imaging is a helpful tool in evaluating the vascularization of focal or diffuse thyroid pathologies. Volumetric analysis is essential for size estimation in cases with disturbed function and for follow up.

US elastography is gaining popularity, especially in the diagnostic of thyroid nodules [8]*.* This method allows a noninvasive evaluation of elasticity (mechanical properties) in different types of tissue. One can distinguish two main types of elastography used in the thyroid diagnostic: shear wave elastography and strain elastography [9]. In the latter the relative tissue hardness is determined by differences in the sound propagation in non-compressed and compressed tissue. The compression is performed by the investigators probe or by the blood vessels (pulsation of the carotid artery). Compression may be displayed on a scale of 0–6 and should ideally be 3–4. Tissue stiffness is visualized on a scale, where soft tissue areas red, hard tissue areas are blue, intermediate yellow and green, respectively. In addition to a color representation, the quantitative measurement of the compressibility of a defined area is also available as percentage value, or strain ratio [10]. Shear-wave elastography measures a mechanical or acoustic wave emitted into the tissue and the propagation speed of shear waves in the tissue. The propagation speed increases with the tissue stiffness. Some methods measure the tissue hardness in a selectable "region of interest" of mostly 5 × 5 mm, others measure this distributed over the parenchyma at every location. Depending on the method, the propagation speed is either specified in m / s or the stiffness using a formula made up of tissue density and propagation velocity calculated in kPa. Measurement of the shear wave speed allows not only qualitatively but also quantitatively estimate the tissue elasticity [10]*.*

CEUS is another novel US technique that is used to investigate dynamic micro vessel perfusion of focal thyroid lesions and thus better characterize and differentiate thyroid nodules [11]*.* This method involves bolus intravenous administration of contrast agent (SonoVue®, Bracco SpA, Milan) which contains sulfur hexafluoride gas microbubbles that are smaller than erythrocyte. Due to the US scattering effect produced by blood capillary, it can visualize the blood perfusion features of thyroid lesions [12]. However, very few studies have been published on the use of CEUS regarding diagnostic of thyroid lesions in children, probably partly due to its “off-label” nature. Even the recent EFSUMB Position Statement does not mention the use of CEUS in the diagnostic of thyroid pathology [13].

### Congenital thyroid abnormalities

Congenital hypothyroidism affects nearly 1 in 2.000 neonates and its early diagnosis is crucial for the neurocognitive development [14]*.* Congenital hypothyroidism can be caused by either dyshormogenesis or by thyroid dysgenesis. Thyroid dysgenesis can present with glandular aplasia, hypoplasia or ectopic gland and it is the most common cause of primary congenital hypothyroidism with an incidence of 1:4.000 [15]. It is usually sporadic and can rarely be familial.

**Fig. 1:**
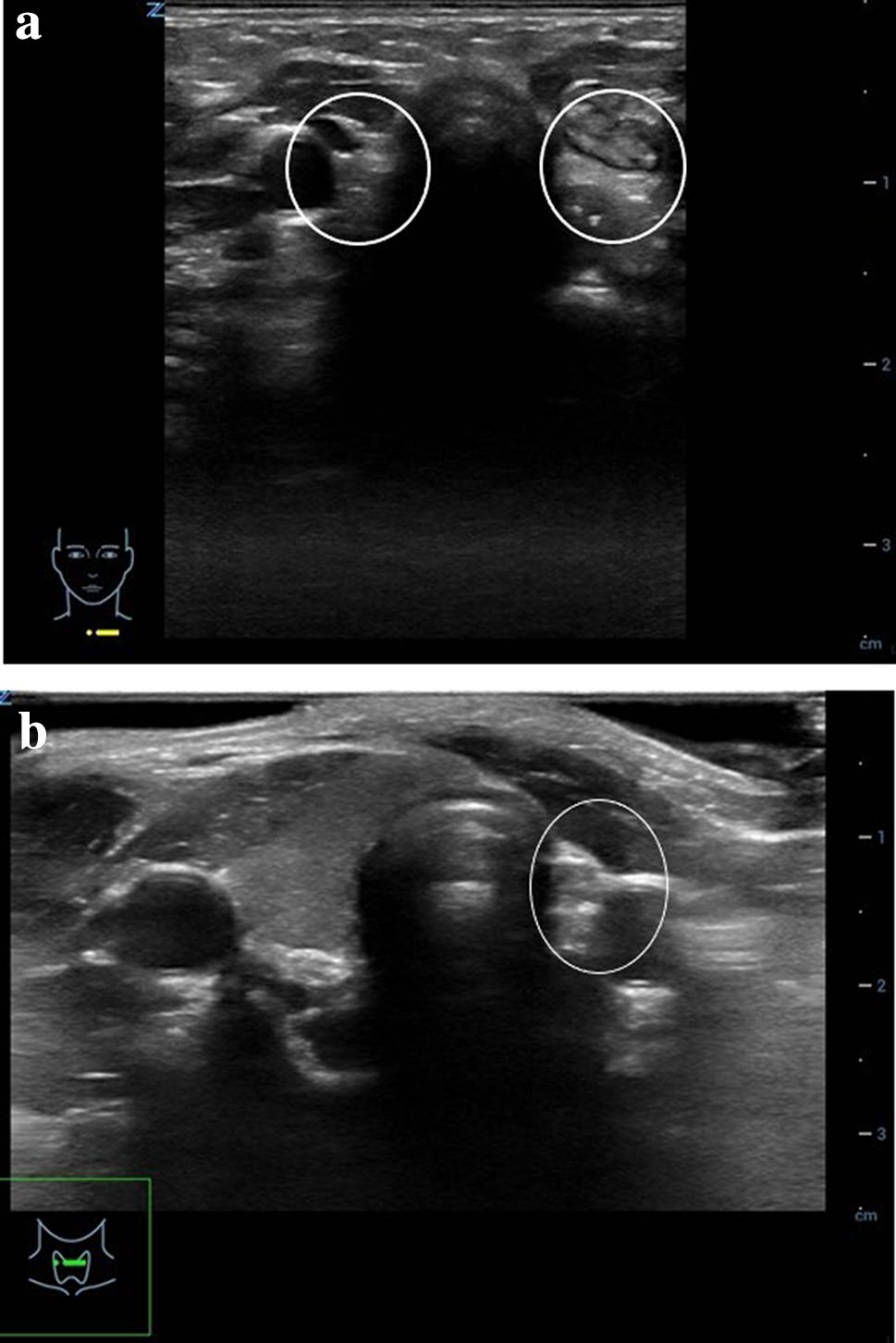
Conventional US images of thyroid anomaly. **a** B-mode US image in a transverse plane shows an aplastic thyroid of a new-born with no evidence of thyroid tissue (circled areas on both sides of the trachea). **b** B-mode US image of a thyroid agenesia of the left lobe in a 7-year-old boy. In a transverse plane right thyroid lobe shows normal echogenicity and is not enlarged. Hyperechogenic structure (circled area) lateral left to the trachea represents remnants of connective tissue

Sonography can differentiate various types of congenital hypothyroidism. In patients with thyroid aplasia the gland cannot be visible neither in the normal position nor along the whole embryologic descent pathway along the thyroglossal duct from the base of the tongue. The small echogenic structures at each side of the trachea represent remnants of connective tissue and ultimate ultimobranchial structures (Fig. 1a).

Scintigraphy is more sensitive for thyroid gland tissue and can be used to confirm the diagnosis of thyroid aplasia or detect even small amounts of functioning thyroid tissue in patients with ectopic thyroid gland [16]. It can be found anywhere along the gland migration course but most often it is located at the base of the tongue [17]. Other studies demonstrate more rare locations such as infra- or suprahyoidal, mediastinal and even lateral cervical [18, 19]*.*

Thyroid hemiagenesis is generally discovered incidentally. Patients demonstrate absent lobe and normal or enlarged contralateral lobe (Fig. 1b).

### Diffuse thyroid disease (DTD)

Most cases of diffuse enlargement of the thyroid gland in children are due to one of the following conditions: chronic lymphocytic thyroiditis (Hashimoto thyroiditis); diffuse hyperplasia (Graves disease), diffuse papillary carcinoma, suppurative thyroiditis and subacute non-suppurative thyroiditis (De Quervain) [20]*.* US can depict findings indicative of DTD, which include diffuse gland enlargement, heterogeneous parenchymal echogenicity, micronodulation, scattered microcalcifications as well as increased or (in later stages) decreased vascularity [16].

#### Hashimoto thyroiditis

This is the most common diffuse thyroiditis which is characterized by lymphocytic infiltration of the thyroid. It affects 1.2% of children and affects predominantly females (4–7:1) [21]*.* Patients are often hypothyroid [22]. It can be associated with Turner syndrome, Noonan syndrome, Down syndrome, phenytoin therapy, juvenile diabetes mellitus and treated Hodgkin disease [22]*.*

The diagnosis is made by detecting antithyroid antibodies (thyroglobulin antibodies and thyroid peroxidase antibodies). These have high sensitivity but low specificity and can be absent in 10–15% of patients [22]*.*

**Fig. 2:**
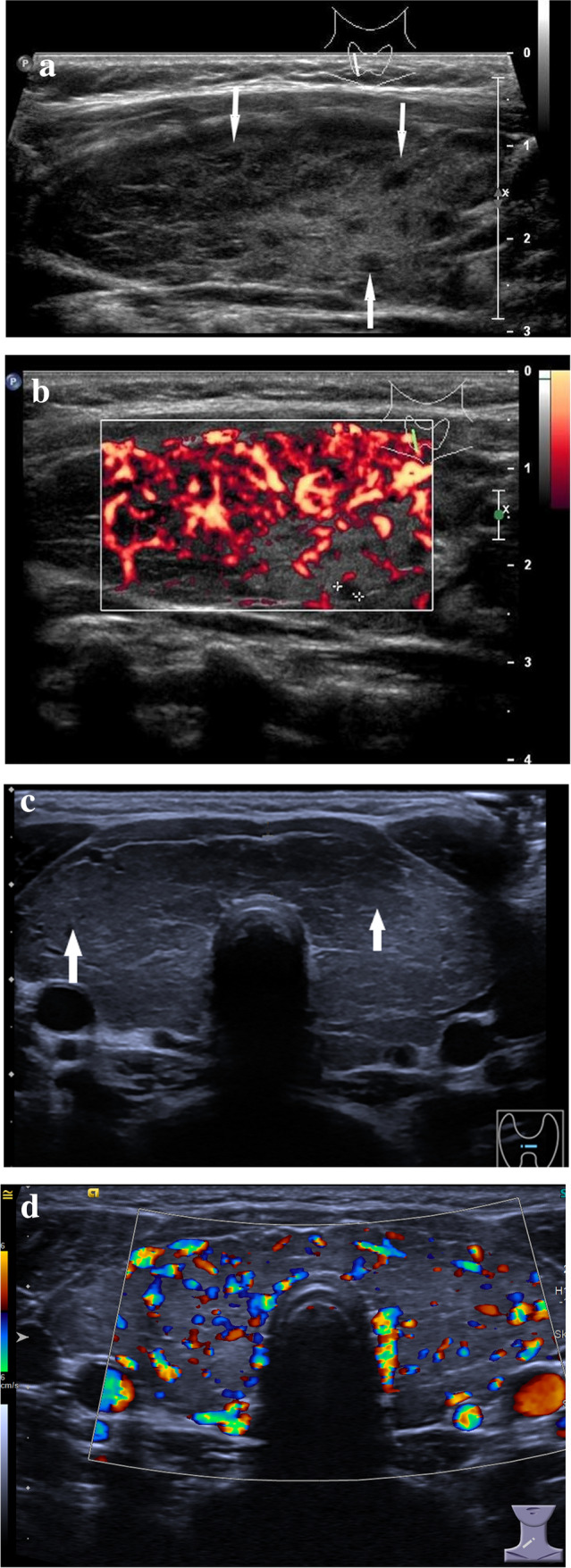
Conventional US and color Doppler US images of diffuse thyroid disease. **a** B-mode US image in a longitudinal plane shows enlarged thyroid with diffuse spreading of hypoechoic lesions (arrows) in a 17-year-old girl with Hashimoto thyroiditis. **b** Color Doppler US image in a longitudinal plane shows increased vascularity in a 17-year-old girl with Hashimoto thyroiditis. **c** B-mode US image in a transverse plane of an enlarged thyroid with hypoechoic areas (arrows) in an 11-year-old girl with Graves’ disease. **d** Color Doppler US image in a transverse plane shows increased vascularity of the thyroid gland in an 11-year-old girl with Graves’ disease referred to as “thyroid inferno”

On US, the gland is enlarged and heterogeneously hypoechogenic due to lymphocytic aggregations (Fig. 2a). It can occasionally present with septations from fibrous bands [16, 23]. Another sonographic pattern of Hashimoto thyroiditis can be pseudo-nodularity with discrete hypoechogenic micronodules 1–6 mm. Vascularity is normal in early stages and increases as disease progresses (Fig. 2b). Later stages are usually associated with decreased vascularity. Lymphatic nodes near by the gland can be reactively enlarged.

#### Graves disease

It is an autoimmune disease which, although rare in children, accounts for the main reason of hyperthyroidism in pediatric population. It is caused by anti-TSH R antibodies that stimulate thyrotropic receptors and cause thyroid growth [24]*.* Lymphocytes and plasmocytes infiltrate the thyroid gland and retro-orbital tissue. That results in patients presenting with an enlarged thyroid gland, exophthalmos, and thyrotoxicosis. There is a strong female predilection (5:1 female-to-male ratio). Graves’ disease is more common in children with other autoimmune diseases such as Type 1 Diabetes, Turner’s Syndrome, Down Syndrome, DiGeorge Syndrome and in children with a family history of autoimmune thyroid disease [25].

On US, the thyroid gland is diffusely enlarged, heterogeneous with hypoechoic echotexture (Fig. 2c) [26]*.* On color Doppler, it presents with strong increased vascularity referred to as “thyroid inferno” (Fig. 2d).

#### Subacute non-suppurative thyroiditis (De Quervain)

It is an inflammatory disorder of the thyroid gland which is rare in adults and even more rare in children [27]*.* Patients present with painful thyroid enlargement usually caused by a viral infection in genetically predisposed individuals.

**Fig. 3:**
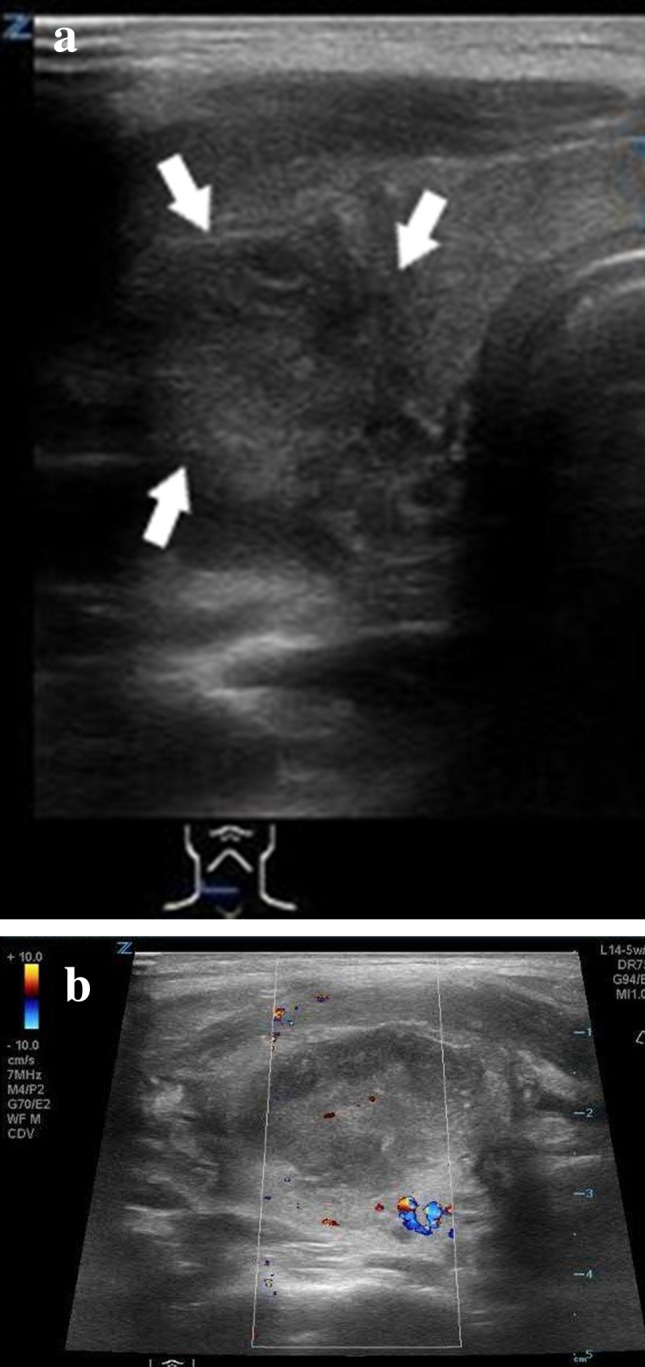
Conventional US and color Doppler US images in a 18-year-old male suffering from local pain, difficulties swallowing, fever. **a** B-mode US image in a transverse plane shows enlarged hypoechoic right lobe of the thyroid gland (arrows) in a patient with subacute non-suppurative thyroiditis. **b** Color Doppler US image in a longitudinal plane shows no vascular flow in a patient with subacute non-suppurative thyroiditis

On US one can confirm the gland enlargement with hypoechoic appearance (Fig. 3a,b). It also demonstrates low to normal vascular flow on Color Doppler and decreased elasticity on elastography [27, 28]*.*

### Focal thyroid lesions

The American thyroid Association (ATA) defines thyroid nodules as “discrete lesions within the thyroid gland, radiologically distinct from surrounding thyroid parenchyma” [29].Thyroid nodules are less common in children than in adults but more likely to be malignant [30, 31]. Thus, following clinical examination and laboratory evaluation, US plays an important role in the diagnostic algorithm of the thyroid nodules in children [29]. Sonographic report should include the description of thyroid nodule shape, composition (solid, cystic, or spongiform), margins (smooth, irregular, lobulated, ill defined, halo extrathyroid extension), echogenicity (hyper-, iso-, hypoechoic), presence/absence of classifications, vascularity, and presence/absence of suspicious cervical lymph adenopathy. Based on the sum of the certain US points, mentioned above, American College of Radiology (ACR) has established a scoring system (TI-RADS) in adults, which corresponds to a risk level ranging from TR1 (benign) to TR5 (highly suspicious), which, in conjunction with the nodule’s maximal dimension, is used to guide management [32]. Unfortunately, larger study performed in children show that ACR TI-RADS is insufficient for evaluation of nodules in this age group and certain US criteria should be modified in order not to miss thyroid cancer in pediatric population [32].

#### Benign thyroid lesions: thyroid cysts

**Fig. 4:**
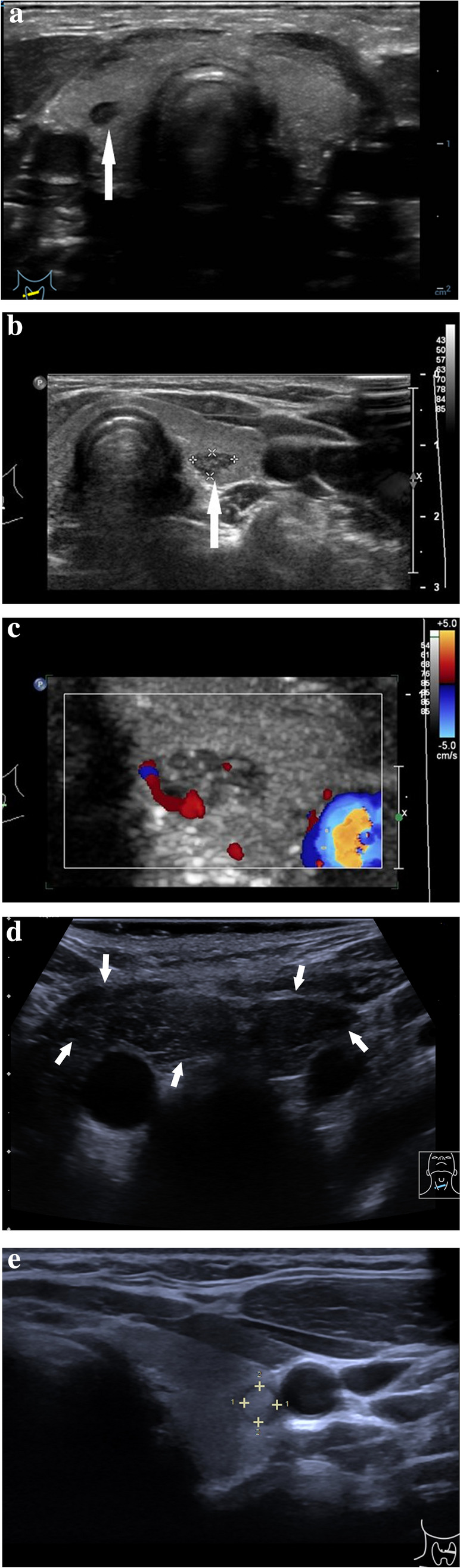
Conventional US and color Doppler US images of benign thyroid lesions. **a** B-mode US in a transverse plane shows colloid cyst (arrow) in a newborn of a mother with known history of Graves’ Disease. **b** B-mode US image in a transverse plane shows an incidentally found intrathyroidal hypoechoic lesion (arrow) with a starry sky pattern in a 7-year-old boy. **c** Color Doppler US image in a transverse plane shows little to no vascular flow in a 7-year-old boy with an incidentally found intrathyroidal lesion. **d** B-mode US image in a transverse plane of the mediastinal thymus (arrows)in a 7-year-old boy shows an identical starry sky pattern, consistent with the normal sonographic appearance of thyroid tissue. **e** B-mode US image in a transversal plane shows an incidentally found intrathyroidal hyperechoic lesion with hypoechoic borders in a 13-year-old boy, consistent with the sonographic appearance of a thyroid adenoma

The majority of pediatric nodules are benign and represent cysts [33]. Most cystic thyroid lesions are hyperplastic nodules that have undergone extensive degeneration; true epithelial lined thyroid cysts are rare. On US, benign cystic lesions are characterized by an echogenic dot and posterior comet tail artifact, which is caused by the presence of microcrystals [34]. Depending on their size, cystic lesions ≤ 0.3 cm are referred to as colloid follicles, whereas cystic lesions from 0.3 to 1 cm are referred to as colloid cysts (Fig. 4a). On color Doppler, true cysts show no vascularity.

#### Benign thyroid lesions: ectopic thymus

The prevalence of ectopic thymus in the thyroid gland varies in the literature from 0,4% to 17,3% [35, 36]. Ectopic thymus tissue is caused by an aberrant thymic migration during embryogenesis and is usually found incidentally. The most common location of ectopic thymus was in the inferior aspect of the left thyroid lobe [36]. The lesion is hypovascular, with angulated borders and has the same US pattern (“starry sky”) as the patients’ mediastinal thymus [37] (Fig. 4b–d).

#### Benign thyroid lesions: thyroid adenomas

Thyroid adenomas are benign lesions of the thyroid gland. These lesions may be inactive or active, producing thyroid hormones. Inactive thyroid adenomas are referred to as follicular adenomas and represent common neoplasms of the thyroid gland [38]. Suppressed TSH—levels suggest a hyperfunctioning thyroid nodule. In this case, it may be referred to as autonomous thyroid nodule or toxic thyroid adenoma. Toxic adenomas are frequently associated with somatic mutations in their TSH receptor or G_s-_ α subunit genes [39]. In addition to US, radionuclide study can be performed because hyperfunctioning thyroid nodules turn to be DTC in one third of pediatric population [40]. Sonographic features that are associated with a higher risk of malignancy are hypoechogenicity, microcalcifications, irregular margins, absent halo sign, and increased intramodular blood flow [41]. Studies show that hard score on elastography is associated with reduced/suppressed TSH [42]. Thus, in patients with hard thyroid nodules on US the presence of autonomously functioning thyroid nodule should be considered (Fig. 4e).

#### Benign thyroid lesions: nodular/diffuse goiter

**Fig. 5:**
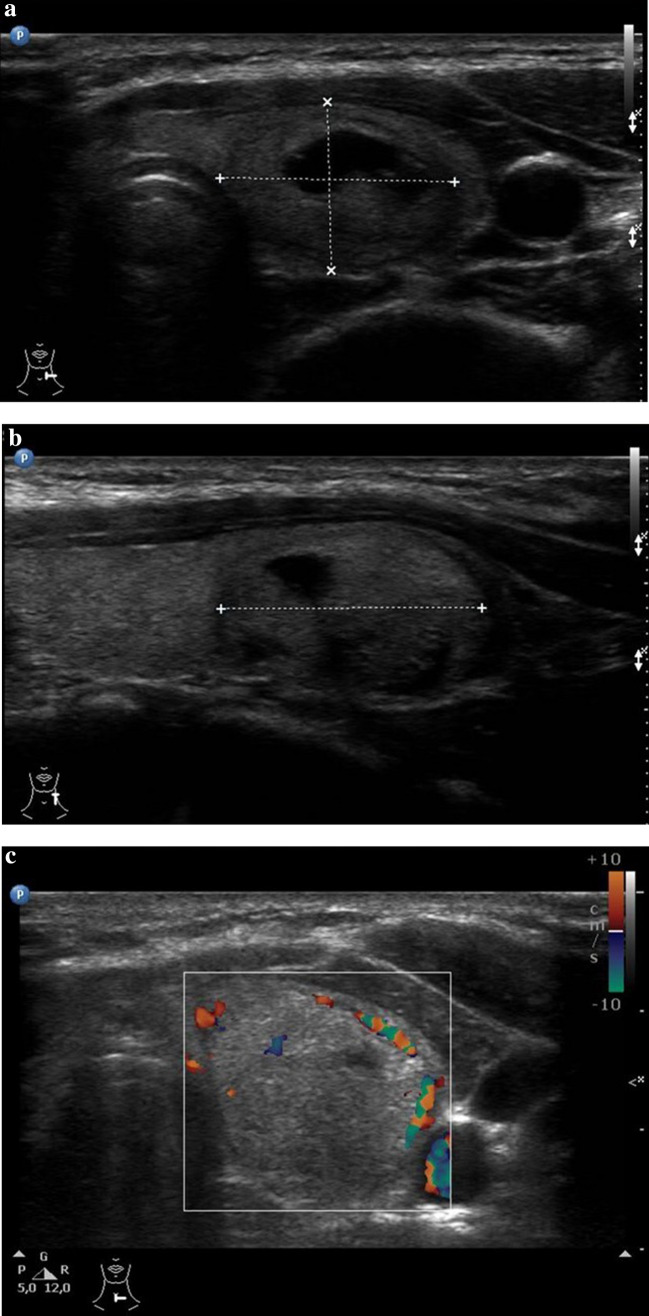
Conventional US and color Doppler US images of benign thyroid lesions. **a** B-mode US image in a transversal plane of the left thyroid lobe shows a partially fluid filled regressive lesion with a mixed echo structure and hypoechoic borders in a 5-year-old girl with nodular goiter. **b** B-mode US image in a longitudinal plane of the left thyroid lobe shows a slightly hyperechoic, partially fluid filled regressive lesion with hypoechoic borders in a 5-year-old girl with nodular goiter. **c** Color Doppler US image in a transverse plane shows little vascular flow in a thyroid lesion in a 5-year-old girl with nodular goiter

Goiter is characterized by a diffuse or localized enlargement of the thyroid at any stage of life. Epidemiologically one can distinguish sporadic or endemic forms, the latter being present in more than 10% of the population due to chronic iodine deficiency [43]. In patients with goiter the gland can either be uniformly enlarged, called diffuse goiter or it can present with one or more nodules within the gland, referred to as nodular or adenomatous goiter. Nodular goiter, in turn, can present with either active autonomous nodules (toxic goiter) or inactive thyroid nodules (non-toxic goiter) [44].Nodules may be solid, filled with fluid, or partly fluid and partly solid) (Fig. 5a–c). US characteristics of benign nodular goiter include presence of a halo, defined borders, absence of microcalcifications, absence of central vascular flow and absence of cervical lymphadenopathy [43]. It is important to remember, however, that each thyroid nodule carries an independent risk of cancer development [45]. That is why US evaluation may indicate the need to complement the assessment with a cytological analysis [46].

#### Intermediate and suspicious malignant lesions

Thyroid lesions are considered to be intermediate when they demonstrate one or more of the following sonographic criteria: slightly isoechoic or hypoechoic structure, with ovoid-to-round shape and smooth or ill-defined margins; intranodular vascularization, elevated stiffness on elastography and presence of macro- or microcalcifications [47].

**Fig. 6:**
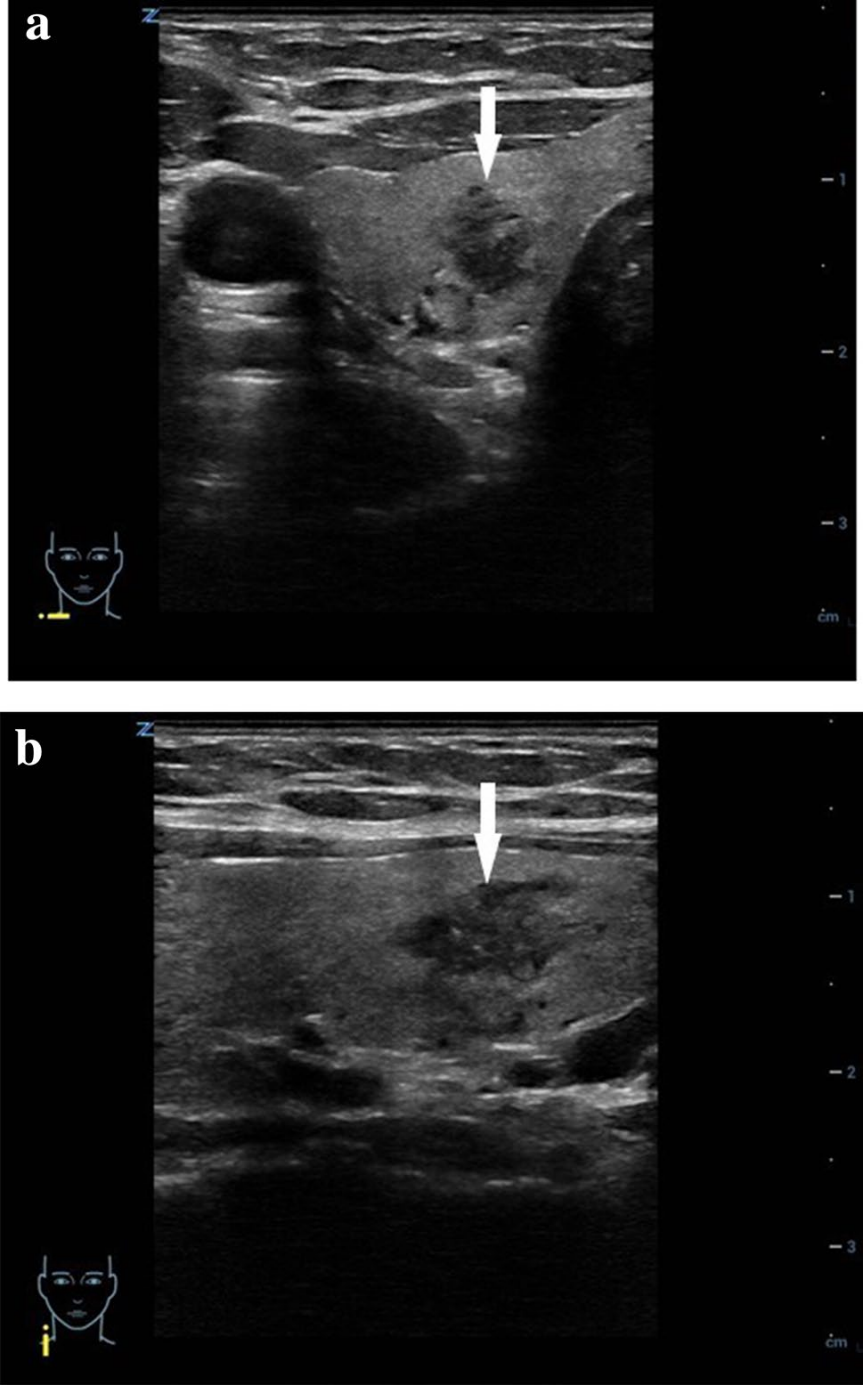
Conventional US images of a suspicious thyroid lesion in a 17-year-old girl. **a** B-mode US image in a transverse plane of the right thyroid gland with an inhomogeneous, predominantly hypoechoic lesion (arrow) with blurry margins which histologically has proven to be papillary carcinoma. **b** B-mode US image in a longitudinal plane of the right thyroid gland with an inhomogeneous, predominantly hypoechoic lesion (arrow) with blurry and partially spiculated margins which histologically has proven to be papillary carcinoma

Thyroid lesions are considered to be suspicious when they demonstrate at least one of the following US criteria: hypoechogenicity, spiculated or microlobulated margins, taller-than-wide shape, microcalcifications, anterior subcapsular location, chaotic vascularity, evidence of extrathyroidal growth or cervical lymphadenopathy [47, 48]. (Fig. 6a, b).

#### Thyroid malignancy

Differentiated thyroid cancer (DTC) most commonly presents as a thyroid nodule. And even though malignant thyroid nodules are rarer in children compared to adults, malignancy rates are higher in pediatric population [31]. The most common type of thyroid malignancy in children is papillary carcinoma (80%), followed by follicular (17%) and medullary (3%) carcinoma, respectively. Two latter types account for less than 10% of pediatric thyroid cancer; medullary type being often diagnosed in patients with multiple endocrine neoplasia syndrome [16].

There have been described certain risk factors for pediatric thyroid cancer in children which include history of thyroid disease, family history of thyroid nodules or thyroid cancer, radiation exposure, radiotherapy, iodine deficiency, elevated serum TSH levels and several genetic syndromes [29, 49].

When diagnosed early, thyroid cancer in children has an excellent prognosis[41]. That is why it's early diagnostic is of crucial importance. Elastography and CEUS can provide additional information and better characterize intermediate or suspicious thyroid lesions prior to fine needle aspiration (FNA) [50].

Application of strain or shear-wave elastography in order to characterize thyroid nodules is based on the principle that benign lesions are usually more elastic than malignant lesions [51]. A baseline strain index for healthy pediatric thyroid tissue has been determined and it shows the value of 0.54 ± 0.38 m/s [52].

The mean shear wave velocity and elasticity values of healthy thyroid in children have also been described in the literature. These values vary from 1.45 ± 0.21 m/s and to 1.82 ± 0.3 m/s and from 6.38 ± 1.97 kPa to 14.6 ± 3.3 kPa, respectively [3, 53, 54]. One recent study in pediatric patients has suggested that high elasticity of a nodule is associated with a low risk of thyroid cancer [55].

**Fig. 7:**
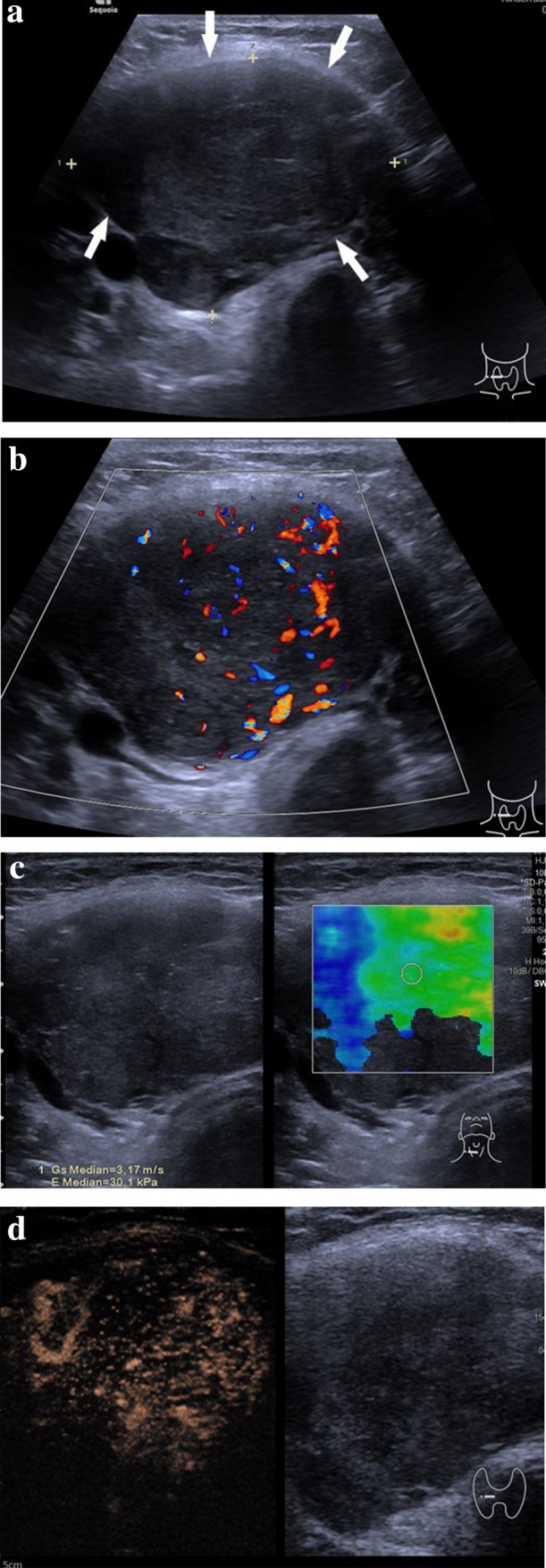
Conventional US, color Doppler US, strain elastography and CEUS images of a malignant thyroid nodule in a 15-year-old boy. **a** B-mode US image of an inhomogeneous slightly isoechoic to hypoechoic lesion (arrows) with ovoid-to-round shape and ill-defined margins; in the right lobe in the longitudinal section. Surgical pathology proved a medullary carcinoma. **b** Color Doppler US image in a transverse plane showing presence of vascularity in a malignant thyroid nodule in the right lobe. **c** SE image of a lesion in a transverse plane showing shear wave elastography (SWE) with increased stiffness expressed as elasticity of 30.1 kPa or high shear wave velocity of 3.17 m/s, indicating a lesion with an elevated stiffness compared to the normal thyroid parenchyma. **d** CEUS image in a transverse plane showing a mass with an uneven distribution of the contrast agent in the lesion indicating heterogeneous enhancement pattern

To better characterize thyroid nodules, CEUS can provide additional information. There have been published various papers regarding CEUS application in thyroid diagnostic in adults, however only one recent study has been published about its application in thyroid diagnostic in children [56]. Due to the US scattering effect produced by blood capillary, CEUS can estimate the blood perfusion features of thyroid nodules to evaluate the angiogenesis situation [12]. The tortuosity and irregularity of newly formed vessels in malignant lesions may lead to heterogeneous enhancement pattern [6] (Fig. 7a–d). Heterogenous enhancement in CEUS can result from a combination of several factors such as neoangiogenesis in the lesion with consecutive hyperenhancement on the one hand and necrosis, interstitial fibrosis, possible calcifications inside the lesion which may all lead to the hypoenhancement on the other hand [57].

## Conclusion

US plays an important role in evaluating thyroid disease in pediatric patients. Application of various US techniques in combination can provide better diagnostic accuracy, help better evaluate thyroid pathology and especially identify suspicious or malignant lesions.

## Abbreviations

ACR: American College of radiology
ATA: American Thyroid Association
B-Mode: Brightness mode
CEUS: Contrast enhanced ultrasound
cm: Centimeter
DTC: Differentiated thyroid cancers
DTD: Diffuse thyroid diseases
EFSUMB: European Federation of Societies in Ultrasound and Medicine
Fig: Figure
FNA: Fine needle aspiration
kPa: Kilopascal
MHz: Megahertz
mm: Millimeters
m/s: Meters per second
TI-RADS: Thyroid imaging reporting and data system
TSH: Thyroid stimulating hormone
TSH R: Thyroid stimulating hormone receptor
US: Ultrasonography

## Declarations

### Statements and Declarations

All data generated or analyzed during this study are included in this published article [and its supplementary information files].
